# Psychometric Properties of the General Organizational Index (GOI): A Measure of Individualization and Quality Improvement to Complement Program Fidelity

**DOI:** 10.1007/s10488-020-01025-2

**Published:** 2020-02-27

**Authors:** Kristin Sverdvik Heiervang, Karina Myhren Egeland, Matthew Landers, Torleif Ruud, Inge Joa, Robert E. Drake, Gary R. Bond

**Affiliations:** 1 Division of Mental Health Services, Akershus University Hospital, Sykehusveien 25, 1478 Lørenskog, Norway; 2 Centre for Medical Ethics, Institute of Health and Society, Medical Faculty, University of Oslo, Oslo, Norway; 3 Duke University, Durham, USA; 4 Institute of Clinical Medicine, University of Oslo, Oslo, Norway; 5 TIPS – Network for Clinical Research in Psychosis, Stavanger University Hospital, Stavanger, Norway; 6 Network for Medical Sciences, Faculty of Health, University of Stavanger, Stavanger, Norway; 7 Westat, Lebanon, NH USA

**Keywords:** GOI general organizational index, Psychometric properties, Individualization and quality improvement

## Abstract

To assess the implementation of effective practices, mental health programs need standardized measures. The General Organizational Index (GOI), although widely used for this purpose, has received minimal psychometric research. For this study, we assessed psychometric properties of the GOI scale administered four times over 18 months during the implementation of a new program in 11 sites. The GOI scale demonstrated high levels of interrater reliability (.97), agreement between assessors on item ratings (86% overall), internal consistency (.77–.80 at three time points), sensitivity to change, and feasibility. We conclude that the GOI scale has acceptable psychometric properties, and its use may enhance implementation and research on evidence-based mental health practices.

**Trial registration:** REK2015/2169.** ClinicalTrials.gov Identifier:** NCT03271242

**Electronic supplementary material:**

The online version of this article (10.1007/s10488-020-01025-2) contains supplementary material, which is available to authorized users.

Successful implementation of an evidence-based practice requires assessment and monitoring of quality (Bond et al. 2011; Martinez et al. 2014; McHugo et al. 2007). The General Organizational Index (GOI) measures two essential components of implementation: individualization and quality improvement (McHugo et al. 2007). Individualization denotes the tailoring of interventions to meet each client’s needs, values, goals, and choices (Sackett et al. 1996). Quality improvement in health care is based on a principle of organizations and staff continuously striving to improve their work. There is no single definition, but it is generally understood to be a systematic approach for improving care and patient outcomes (Ross and Naylor 2017).

Activities and structures documented to improve quality within mental health services are training and education of staff to build competence, ongoing clinical supervision to ensure clinical skill, and commitment at organizational levels such as process monitoring, patient outcome monitoring, and quality assurance to evaluate and improve services (Aarons et al. 2011; Bond et al. 2009a; Egeland et al. 2019; Fixsen et al. 2005; Marty et al. 2008; McGuire et al. 2015; Monroe-DeVita et al. 2012; Rapp et al. 2010; Torrey et al. 2003).

Quality improvement can improve clients’ outcomes (Becker et al. 2007; Rapp et al. 2008; Shannon et al. 2001; Taylor 1987). Furthermore, ongoing supervision, process and outcome monitoring can promote long-term sustainability of an evidence-based practice (Bond et al. 2014; Moullin et al. 2019).

The gap in quality of mental health care is partly due to a lack of systematic methods for measuring quality and quality improvement (Kilbourne et al. 2018). Most developed scales measure quality at the program level by assessing *fidelity* toward a practice using objective data from a clinical team or program on the implementation of key components of the evidence-based model (Bond and Drake 2019). Fidelity is defined as the degree to which a program implementing an evidence-based practice adheres to specific model standards (Bond et al. 2000).

Fidelity assessment of specific practices, however, does not necessarily include measures of individualization of services or quality improvement thought to influence outcomes. These two dimensions go beyond the specific interventions being implemented (Bond et al. 2009a, b) Thus, the efforts by an organization to provide the necessary means and procedures for high quality and sustainable implementation in a broader sense are not usually covered. The GOI scale addresses several of these features.

Researchers have rarely reported the psychometric properties of the GOI scale, despite wide usage (Egeland et al. 2017; McHugo et al. 2007; Salyers et al. 2009). One previous psychometric study of the GOI found acceptable interrater reliability, internal consistency, and sensitivity to change across several evidence-based practices (Bond et al. 2009a, b).

The purpose of this study was to examine the psychometric properties of the GOI scale, including interrater reliability, interrater item agreement, internal consistency, sensitivity to change, and feasibility.

## Methods

### Overview

As part of a large implementation study (Clinical Trials NCT03271242), the research team invited mental health clinics providing treatment for psychosis disorders throughout Norway to participate. Eleven sites from six of the 19 health trusts in Norway agreed to implement Illness Management and Recovery services (Mueser et al. 2006) and received intensive technical assistance and implementation support. This sub-study assessed use of the GOI scale in these 11 sites. The Regional Committees for Medical and Health Research Ethics (REK 2015/2169) approved the study, which followed the principles of the Declaration of Helsinki.

### Study Sites

Of the 11 mental health clinics, eight were community mental health centers, one was a combined inpatient and outpatient clinic for young adults with psychosis and drug abuse problems, one was an outpatient clinic for children and adolescents, and one was an inpatient clinic for adolescents (age 16 and older). The participating clinics represented urban and rural areas.

### Procedures

Illness Management and Recovery trains people with serious mental illness to manage their illness and achieve personal recovery goals (Mueser et al. 2006). The standardized psychosocial intervention contains five elements: psychoeducation to improve knowledge of mental illness; relapse prevention to reduce relapses and rehospitalization; behavioral training to improve medication adherence; coping skills training to reduce the severity and distress of persistent symptoms; and social training to strengthen social support.

Each clinic received intensive technical assistance on the intervention over 12 months, including four days of training with a professional trainer, followed by a 30-min weekly group supervision session by phone for six months, and then group supervision every other week for another six months. In addition, a supervisor visited each site biweekly for the first six months and monthly for the following six months to support leaders and clinicians in monitoring progress by the use of feedback from GOI and fidelity measures, defining goals and strategies, identifying barriers, and solving problems.

At each site, a pair of fidelity assessors who were independent of the clinical staff delivering Illness Management and Recovery conducted a fidelity assessment at baseline, and at 6, 12, and 18 months. Fidelity assessors also completed a GOI assessment during these site visits. The same pair of assessors collected and rated the two scales.

The fidelity assessors varied across sites and assessment periods. A group of 17 researchers (psychologists, psychiatrists, nurses, and other health professionals) served as the assessors. All received specific training on procedures for assessing the GOI.

The assessors conducted full-day site visits, using a combination of four sources of information: (a) semi-structured interviews with the site leader; (b) semi-structured group interviews with practitioners; (c) progress notes on the patients’ goals and progress; and (d) handouts and written materials. After each visit, the two assessors rated each site independently and then compared ratings, resolving discrepancies through discussion to reach consensus.

### Measures

#### GOI Scale

The 12-item GOI scale comprises two subscales: Individualization and Quality Improvement. The two subscales have been confirmed through factor analysis (Bond et al. 2009a, b). The Individualization sub-scale includes five items: *Eligibility*, *Assessment*, *Individualized treatment plan*, *Individualized treatment*, and *Client choice*. The Quality Improvement sub-scale also includes five items: *Training*, *Supervision*, *Process monitoring*, *Outcome monitoring*, and *Quality assurance.* Additional items include *Program philosophy* and *Penetration*.

Ratings on behaviorally anchored scales range from 1, indicating poor implementation, to 5, indicating full implementation. A summed and averaged score of 4.0 or higher can be defined as adequate, 3.0–4.0 as fair and less than 3 as poor. For example, a score of 1 on the item *Training* indicates that ≤ 20% of practitioners received standardized training annually, and a score of 5 indicates that > 80% of practitioners received standardized training annually (see “Online Appendix” section).

The assessment involves a 1-day site visit by two trained fidelity assessors to gather information from various sources in order to make ratings on the 12 items. Assessors follow a protocol with instructions for data collection and scoring procedures.

A Norwegian translation agency translated the GOI scale into Norwegian, in conjunction with the translation of the Illness Management and Recovery manual (Egeland 2018). Two of the authors (KME and KSH) reviewed the translations in detail, repeatedly comparing it with the original version.

After the final GOI assessments, the fidelity assessors completed an online survey on the ease of finding the information, the ease of rating the item when the information was available, and the usefulness of different sources of information and the rating instructions.

### Data Analyses

We examined agreement between assessors at the item level by percentage of exact agreement between pairs of assessors. We also examined mean agreement across items at each time and across all four time points.

We calculated each assessor’s total GOI score for each site, defined as the sum of the item ratings divided by the number of items. To evaluate interrater reliability of the site ratings, we used the Intraclass Correlation Coefficient (McGraw and Wong 1996), based on a one-way random effects analysis of variance model (“average method”) for the GOI scale and the two subscales. A single coefficient combined paired ratings across all assessment points.

After assessing interrater agreement and reliability, we used consensus ratings in all subsequent analyses. To estimate internal consistency of the GOI scale and the two subscales, we calculated Cronbach’s alpha for each time point. We also examined correlations between the two GOI subscales and the total GOI score and with the Illness Management and Recovery Fidelity Scale at each time point.

We next examined the item distributions at 18 months, examining means, standard deviations, and distribution of scores across sites for full (rating = 5), adequate (4), and poor (1–3) scores. We also examined the distribution of site scores at 18 months. Finally, we examined the longitudinal pattern of GOI and the two subscales graphically and statistically using a one-way analysis of variance repeated measures design with post hoc *t*-test comparisons between baseline and each of the three follow-up assessments. Change over time was estimated by calculating the standardized mean difference effect size (Cohen’s dz) for within-subjects design (Lakens 2013). We interpreted the sensitivity to change as adequate if the improvement was statistically significant and with at least a moderate effect size (Cohen’s d $\ge$ 0.50) (Streiner et al. 2015).

We analyzed feasibility using descriptive statistics and paired sample *t*-tests for differences. All data analyses were performed using SPSS software (v. 25; IBM SPSS, Armonk, NY, USA).

## Results

### Agreement Between Assessors on Individual Items

Over all items and time points, exact agreement on items averaged 86%, as shown in Table 1. The mean exact agreement declined from 98% at baseline to 88%, 77%, and 82% thereafter. High agreement at baseline confirmed the lack of implementation of the new practice.

**Table 1:** The GOI: Item agreement between fidelity assessors

Description	IMR (n = 11 sites)				
	Baseline (%)	6 months (%)	12 months (%)	18 months (%)	Mean (%)
G1. Program philosophy	82	100	82	82	86
G2. Eligibility/client identification	100	91	73	91	89
G3. Penetration	100	91	82	82	89
G4. Assessment	100	82	82	73	84
G5. Individualized treatment plan	100	91	82	82	89
G6. Individualized treatment	100	91	91	91	93
G7. Training	100	82	82	82	86
G8. Supervision	100	82	64	82	82
G9. Process monitoring	100	82	55	64	75
G10. Outcome monitoring	100	82	73	91	86
G11. Quality assurance	100	100	91	82	93
G12. Client choice regarding services	100	82	64	82	82
Total scale	98	88	77	82	86

### Interrater Reliability

Two fidelity assessors rated the GOI scales on four occasions at each of the 11 sites implementing Illness Management and Recovery. We aggregated the paired ratings across all four time points to estimate interrater reliability for the 44 assessments (100% completion rate). The Intraclass Correlation Coefficient measuring interrater reliability was 0.97 for GOI total, 0.97 for Individualization, and 0.93 for Quality Improvement. For all subsequent analyses, we used consensus ratings.

### Internal Consistency

Table 2 shows internal consistency (Cronbach’s alpha) for GOI was acceptable: baseline = undefined, 6 months = 0.77, 12 months = 0.80, 18 months = 0.78, and combined = 0.90. Internal consistency at baseline could not be calculated because nearly all items were rated 1 at all sites. The mean internal consistency coefficients were 0.87 for Individualization and 0.76 for Quality Improvement.

**Table 2:** Internal consistency and correlations between the GOI total scale, GOI subscales, and the fidelity scale at 6, 12, and 18 months

Time	Internal consistency			Concurrent correlations			
	Ind^a^	QI^b^	GOI tot	Ind with QI	Ind with IMR fidelity	QI with IMR fidelity	GOI with IMR fidelity
Baseline	No var	No var	0.00	No var	No var	No var	No var
6 months	0.82	0.56	0.77	0.40	0.67*	0.17	0.63*
12 months	0.64	0.40	0.80	0.70*	0.73*	0.35	0.56
18 months	0.81	0.61	0.78	0.18	0.60*	0.21	0.53
Combined (the 3 time periods)	0.87	0.76	0.90	Mean = .43	Mean = .67	Mean = .24	Mean = .57

^a^Individualization

^b^Quality improvement

*Correlation is significant at the 0.05 level (2-tailed)

### Correlations Between GOI Subscales and Illness Management and Recovery Fidelity

Correlations at each time point between the two subscales ranged from 0.18 to 0.70 (Table 2). Mean correlation between the two scales for all three time points was 0.43. Correlations between the GOI scale and the Illness Management and Recovery Fidelity Scale ranged from 0.53 to 0.63 (mean correlation = 0.57).

### Item Analysis

In Table 3 we show the item distributions, including the number of sites achieving poor, adequate, and full fidelity. On only four items did a majority of programs achieve adequate or full fidelity scores (Item 1: *Program philosophy*, Item 6: *Individualized treatment*, Item 9: *Process monitoring*, and Item 12: *Client choice*). Thus the item analysis shows a number of areas needing improvement.

**Table 3:** Item distributions for the GOI 18 months after start-up

Item	Description	N = 11	GOI item ratings by site		
		Mean (SD)	Low	Adequate	Full
Individualization					
2	Eligibility/client identification	2.45 (1.63)	8	1	2
4	Assessment	3.45 (1.63)	6	0	5
5	Individualized treatment plan	2.82 (1.83)	7	0	4
6	Individualized treatment	3.91 (1.45)	4	1	6
12	Client choice regarding services	4.09 (1.45)	3	1	7
Quality improvement					
7	Training	2.82 (1.83)	6	2	3
8	Supervision	2.55 (1.37)	8	2	1
9	Process monitoring	4.09 (1.14)	1	6	4
10	Outcome monitoring	1.82 (1.25)	9	2	0
11	Quality assurance	2.36 (1.63)	8	1	2
Additional items					
1	Program philosophy	4.55 (0.52)	0	5	6
3	Penetration	1.82 (1.25)	10	0	1
	Mean GOI rating	3.06 (0.79)	9	2	0

### Changes Over Time

**Fig. 1:**
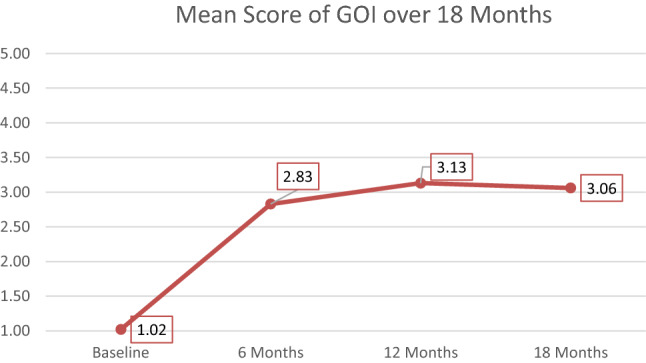
Mean score of the GOI over 18 months

**Fig. 2:**
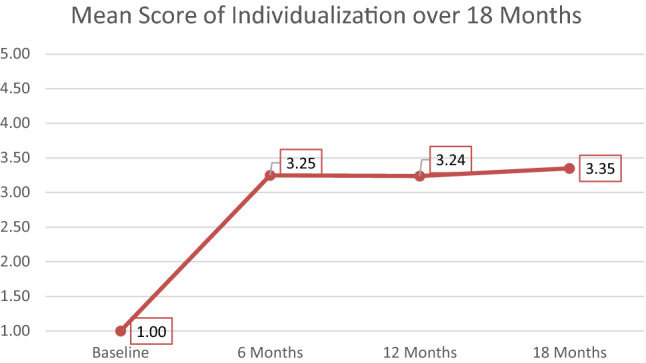
Mean score of Individualization over 18 months

**Fig. 3:**
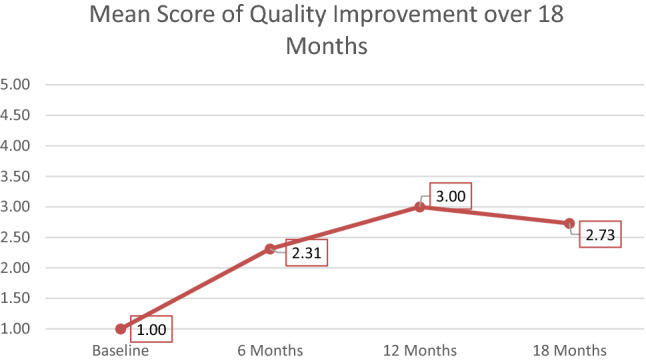
Mean score of Quality Improvement over 18 months

We visually inspected the graphical longitudinal pattern of changes across the 18-month period for the 11 sites (Figs. 1, 2 and 3). The mean improvement was sharp between baseline and 6 months and plateaued at 12 and 18 months. Post hoc *t*-tests comparing baseline GOI ratings to 6-, 12-, and 18-month ratings confirmed statistically significant sensitivity to change, with *t* values of 7.02 at 6 months, 8.42 at 12 months, and 8.59 at 18 months (all significant at *p* < 0.001). The standardized mean difference effect size (Cohen’s d_z_) was very large 2.59.

The numbers and percentages of sites attaining adequate GOI (≥ 4.0) were one (9%) at 6 months, two (18%) at 12 months, and two (18%) at 18 months.

### Feasibility

The 17 fidelity assessors assessed an average of 14 sites each (including the sites in the control group) across the four time points. They reported that it was significantly easier to rate the items than to find the information (*t* = 3.61, df = 16, *p* = 0.002). The interviews with clinicians were the most useful sources of information, while observations or other information were moderately useful, and information of written procedures was of little or no use. The instructions were acceptable.

## Discussion

The current study demonstrated that the psychometric properties of the GOI scale were good to excellent, including interrater reliability, agreement between assessors, internal consistency, sensitivity to change, use of the entire rating scale, and feasibility. The GOI scale was moderately correlated with fidelity, suggesting that the GOI scale is measuring dimensions associated with fidelity to evidence-based practices and that adherence to GOI principles may promote fidelity (Bond et al. 2009a, b). Nevertheless, all sites failed to reach high GOI scores in six months, and most sites were still attaining low GOI scores at 12 and 18 months.

Adequate psychometrics should be a sine qua non for a measure of quality. The current study replicates the acceptable psychometric findings of the GOI in one previous study (Bond et al. 2009a, b). These two studies should establish the usability of the GOI scale.

Several studies have found that sites rarely reach high scores on the GOI, even 12, 18, and 24 months after baseline (Bond et al. 2009a, b; Salyers et al. 2009; Egeland et al. 2017). The tension between high standards and attainability pervades the field of implementation (Salyers et al. 2003). The gap could indicate that the scale standards are unrealistic or, alternatively, may reflect that organizational change is difficult. Earlier research has shown that practitioners often reject a commitment to monitoring quality (Bond et al. 2009a, b; Bond et al. 2014; Egeland 2018; Rychener et al. 2009). The site with highest GOI ratings in the current study had strong management, prioritized organizational changes, provided training and supervision to every staff member, offered Illness Management and Recovery to every patient, and established a quality assurance committee with responsibility for implementation and regular review. Thus, implementation of a new practice may depend on unusual levels of commitment.

Refinements of the GOI may require a focus on specific items. The findings identified three items with lower (still adequate) agreement: *Supervision*, *Process monitoring*, and *Client choice*. To improve agreement on these items in future assessments interviews with patients and observations of meetings and supervision, which the current study did not include, are recommended (Bond et al. 2009a, b). Other items may need recalibration if standards are unrealistic.

Some limitations deserve mention. The GOI assessments included neither interviews with patients nor observation of meetings. The current study did not test the validity of the scale. Most critical, it would be useful to know whether using the GOI to ensure Individualization and Quality Improvement improves patient outcomes. No published study has examined the predictive validity of the GOI scale, a critical next step.

## Conclusions and Implications

The GOI scale demonstrates good to excellent psychometric properties in terms of high interrater reliability, good internal consistency, sensitivity to change, and feasibility of use. Consistent with earlier findings, the GOI scale has the required psychometric properties to measure the Individualization and Quality Improvement. A strength of the GOI scale is applicability across multiple practices. Its use should enhance the quality and sustainability of different evidence-based practices.

## Electronic supplementary material

Below is the link to the electronic supplementary material.

Supplementary file1 (DOC 57 kb)
