# Supplementary material for: Psychometric Properties of the General Organizational Index (GOI): A Measure of Individualization and Quality Improvement to Complement Program Fidelity
Source: Adm Policy Ment Health. 2020 Feb 27;47(6):920–6. doi: 10.1007/s10488-020-01025-2 (PMC7547970; doi:10.1007/s10488-020-01025-2)
Supplement: Supplementary file 1 — Supplementary file1 (DOC 57 kb) [file 10488_2020_1025_MOESM1_ESM.doc]

|  | **1** | **2** | **3** | **4** | **5** |
| --- | --- | --- | --- | --- | --- |
| **G1. Program Philosophy.** The program is committed to a clearly articulated philosophy consistent with the specific evidence-based model, based on the following 5 sources:   - Program leader - Senior staff (e.g., executive director, psychiatrist) - Practitioners providing the EBP - Clients and/or families receiving EBP - Written materials (e.g., brochures) | No more than 1 of the 5 sources shows clear understanding of the program philosophy  OR  All sources have numerous major areas of discrepancy | 2 of the 5 sources show clear understanding of the program philosophy  OR  All sources have several major areas of discrepancy | 3 of the 5 sources show clear understanding of the program philosophy  OR  Sources mostly aligned to program philosophy, but have one major area of discrepancy | 4 of the 5 sources show clear understanding of the program philosophy  OR  Sources mostly aligned to program philosophy, but have one or two minor areas of discrepancy | All 5 sources display a clear understanding and commitment to the program philosophy for the specific EBP |
| ***G2. Eligibility/Client Identification.** All clients with severe mental illness in the community support program, crisis clients, and institutionalized clients are screened to determine whether they qualify for the EBP using standardized tools or admission criteria consistent with the EBP. Also, the agency tracks the number of eligible clients in a systematic fashion. | ≤20% of clients receive standardized screening and/or agency DOES NOT systematically track eligibility | 21%-40% of clients receive standardized screening and agency systematically tracks eligibility | 41%-60% of clients receive standardized screening and agency systematically tracks eligibility | 61%-80% of clients receive standardized screening and agency systematically tracks eligibility | >80% of clients receive standardized screening and agency systematically tracks eligibility |
| ***G3. Penetration.** The maximum number of eligible clients are served by the EBP, as defined by the ratio:  **# clients receiving EBP**  **# clients eligible for EBP** | Ratio ≤ .20 | Ratio between .21 and .40 | Ratio between .41 and .60 | Ratio between .61 and .80 | Ratio > .80 |

*These two items coded based on all clients with SMI at the site or sites where the EBP is being implemented; all other items refer specifically to those receiving the EBP.

**________ Total # clients in target population**

# ________ Total # clients eligible for EBP % eligible: ___%

________ **Total # clients receiving EBP Penetration rate: ____**

|  | **1** | **2** | **3** | **4** | **5** |
| --- | --- | --- | --- | --- | --- |
| **G4. Assessment.** Full standardized assessment of all clients who receive EBP services. Assessment includes history and treatment of medical/psychiatric/  substance use disorders, current stages of all existing disorders, vocational history, any existing support network, and evaluation of biopsychosocial risk factors. | Assessments are completely absent or completely non-standardized | Pervasive deficiencies in two of the following:  Standardization,  Quality of assessments, Timeliness, Comprehensive-ness | Pervasive deficiencies in one of the following:  Standardization,  Quality of assessments, Timeliness, Comprehensive-ness | 61%-80% of clients receive standardized, high quality assessments at least annually  OR  Information is deficient for one or two assessment domains | >80% of clients receive standardized, high quality assessments, the information is comprehensive across all assessment domains, and updated at least annually |
| **G5. Individualized Treatment Plan.** For all EBP clients, there is an explicit, individualized treatment plan *related to the EBP* that is consistent with assessment and updated every 3 months. | ≤20% of clients served by EBP have an explicit individualized treatment plan, *related to the EBP*, updated every 3 mos. | 21%-40% of clients served by EBP have an explicit individualized treatment plan, *related to the EBP*, updated every 3 mos. | 41%-60% of clients served by EBP have an explicit individualized treatment plan, *related to the EBP*, updated every 3 mos.  OR  Individualized treatment plan is updated every 6 mos. for all clients | 61%-80% of clients served by EBP have an explicit individualized treatment plan, *related to the EBP*, updated every 3 mos. | >80% of clients served by EBP have an explicit individualized treatment plan *related to the EBP*, updated every 3 mos. |
| **G6. Individualized Treatment.** All EBP clients receive individualized treatment meeting the goals of the EBP. | ≤20% of clients served by EBP receive individualized services meeting the goals of the EBP | 21%-40% of clients served by EBP receive individualized services meeting the goals of the EBP | 41%-60% of clients served by EBP receive individualized services meeting the goals of the EBP | 61% - 80% of clients served by EBP receive individualized services meeting the goals of the EBP | >80% of clients served by EBP receive individualized services meeting the goals of the EBP |

|  | **1** | **2** | **3** | **4** | **5** |
| --- | --- | --- | --- | --- | --- |
| **G7. Training.** All new practitioners receive standardized training in the EBP (at least a 2-day workshop or its equivalent) *within 2 months of hiring.* Existing practitioners receive annual refresher training (at least 1-day workshop or its equivalent). | ≤20% of practitioners receive standardized training annually | 21%-40% of practitioners receive standardized training annually | 41%-60% of practitioners receive standardized training annually | 61%-80% of practitioners receive standardized training annually | >80% of practitioners receive standardized training annually |
| **G8. Supervision.** EBP practitioners receive structured, weekly supervision (group or individual format) from a practitioner experienced in the particular EBP. The supervision should be client-centered and explicitly address the EBP model and its application *to specific client situations.* | ≤20% of practitioners receive supervision | 21% - 40% of practitioners receive weekly structured client-centered supervision  OR  All EBP practitioners receive supervision on an informal basis | 41%-60% of practitioners receive weekly structured client-centered supervision  OR  All EBP practitioners receive supervision monthly | 61%-80% of EBP practitioners receive weekly structured client-centered supervision  OR  All EBP practitioners receive supervision twice a month | >80% of EBP practitioners receive structured weekly supervision, focusing on specific clients, in sessions *that explicitly address the EBP model and its application* |
| **G9. Process Monitoring.** Supervisors and program leaders monitor the process of implementing the EBP every 6 months and use the data to improve the program. Monitoring involves a standardized approach, e.g., use of a fidelity scale or other comprehensive set of process indicators. | No attempt at monitoring process is made | Informal process monitoring is used at least annually | Process monitoring is deficient on 2 of these 3 criteria: (1) Comprehensive & standardized; (2) Completed every 6 mos.; (3) Used to guide program improvements OR  Standardized monitoring done annually only | Process monitoring is deficient on one of these three criteria: (1) Comprehensive and standardized; (2) Completed every 6 months; (3) Used to guide program improvements | Standardized comprehensive process monitoring occurs at least every 6 mos. and is used to guide program improvements |

|  | **1** | **2** | **3** | **4** | **5** |
| --- | --- | --- | --- | --- | --- |
| **G10. Outcome Monitoring.** Supervisors/program leaders monitor the outcomes for EBP clients every 3 months and share the data with EBP practitioners. Monitoring involves a standardized approach to assessing a key outcome *related to the EBP*, e.g., psychiatric admissions, substance abuse treatment scale, or employment rate. | No outcome monitoring occurs | Outcome monitoring occurs at least once a year, but results are not shared with practitioners | Standardized outcome monitoring occurs at least once a year and results are shared with practitioners | Standardized outcome monitoring occurs at least twice a year and results are shared with practitioners | Standardized outcome monitoring occurs quarterly and results are shared with EBP practitioners |
| **G11. Quality Assurance (QA).** The agency has a QA Committee or implementation steering committee with an explicit plan to review the EBP, or components of the program, every 6 months. | No review or no committee | QA committee has been formed, but no reviews have been completed | Explicit QA review occurs less than annually OR  QA review is superficial | Explicit QA review occurs annually | Explicit review every 6 months by a QA group *or steering committee for the EBP* |
| **G12. Client Choice Regarding Service Provision.** All clients receiving EBP services are offered choices; the EBP practitioners consider and abide by client preferences for treatment when offering and providing services. | Client-centered services are absent (or all EBP decisions are made by staff) | Few sources agree that type and frequency of EBP services reflect client choice | Half sources agree that type and frequency of EBP services reflect client choice | Most sources agree that type and frequency of EBP services reflect client choice OR  Agency fully embraces client choice with one exception | All sources agree that type and frequency of EBP services reflect client choice |
